# Homeostasis model assessment, serum insulin and their relation to body fat in cats

**DOI:** 10.1186/s12917-020-02729-1

**Published:** 2021-01-18

**Authors:** Emma M. Strage, Charles J. Ley, Johannes Forkman, Malin Öhlund, Sarah Stadig, Anna Bergh, Cecilia Ley

**Affiliations:** 1 Department of Clinical Sciences, Swedish University of Agricultural Sciences, Box 7054, 750 07 Uppsala, Sweden; 2 University Animal Hospital, Swedish University of Agricultural Sciences, Uppsala, Sweden; 3 Department of Crop Production Ecology, Swedish University of Agricultural Sciences, Uppsala, Sweden; 4 Department of Biomedical Sciences and Veterinary Public Health, Swedish University of Agricultural Sciences, 750 07 Uppsala, Sweden

**Keywords:** Obesity, Glucose, Biological variation, Feline

## Abstract

**Background:**

Obesity is associated with insulin resistance (IR) and considered a risk factor for diabetes mellitus (DM) in cats. It has been proposed that homeostasis model assessment (HOMA-IR), which is the product of fasting serum insulin (mU/L) and glucose (mmol/L) divided by 22.5, can be used to indicate IR. The objectives of this study were threefold: (i) to evaluate associations between body fat, fasting insulin, and HOMA-IR, (ii) to determine population-based reference interval of HOMA-IR in healthy lean cats, and (iii) to evaluate biological variation of HOMA-IR and fasting insulin in cats.

**Results:**

150 cats were grouped as lean or overweight based on body condition score and in 68 of the cats body fat percentage (BF%) was estimated by computed tomography. Fasting serum insulin and glucose concentrations were analysed. Statistical differences in HOMA-IR and insulin between overweight or lean cats were evaluated using Wilcoxon rank-sum test. Robust method with Box-Cox transformation was used for calculating HOMA-IR reference interval in healthy lean cats. Relations between BF% and HOMA-IR and insulin were evaluated by regression analysis. Restricted maximum likelihood ratio was used to calculate indices of biological variation of HOMA-IR and insulin in seven cats.

There were significant differences between groups with overweight cats (*n* = 77) having higher HOMA-IR (*p* < 0.0001) and insulin (*p* = 0.0002) than lean cats (*n* = 73). Reference interval for HOMA-IR in lean cats was 0.1–3.0. HOMA-IR and fasting insulin concentrations showed similar significant positive association with BF% (*p* = 0.0010 and *p* = 0.0017, respectively). Within-animal coefficient of variation of HOMA-IR and insulin was 51% and 49%, respectively.

**Conclusions:**

HOMA-IR and fasting insulin higher in overweight than lean cats and correlate to BF%. The established population-based reference interval for HOMA-IR as well as the indices of biological variation for HOMA-IR and fasting insulin may be used when interpreting HOMA-IR and fasting insulin in cats. Further studies are needed to evaluate if HOMA-IR or fasting insulin is useful for identifying cats at risk of developing DM.

## Background

Diabetes mellitus (DM) is a common metabolic disease in cats [1]. Feline DM is considered pathophysiologically similar to human DM type 2, and is characterized by insulin resistance (IR) and relative insulin deficiency leading to hyperglycemia [1]. Obesity can cause IR [2] and may predispose to development of DM in cats [3]. The most accepted method for evaluating peripheral IR in humans is the euglycemic insulin clamp [4]. This is a labor intensive method seldom performed in veterinary clinical practice. A previous study used simpler methods based on only one blood sample to evaluate IR in cats and concluded that the homeostasis model assessment (HOMA-IR), which is the product of fasting circulating insulin and glucose concentrations divided by 22.5, could be used to screen for IR [5]. A simple method to estimate IR would assist in early identification of insulin resistant cats in clinical practice and allow preventive actions to be taken before these cats develop DM.

When monitoring or screening for IR the individual test result is usually interpreted in relation to a reference interval (RI) derived from healthy animals. Population-based RI of fasting insulin in healthy cats are wide [6] and a previous study of HOMA-IR in ideal weight cats also demonstrated a wide range [5]. However, some analytes present with high variation between animals but little variation within an animal. In these analytes, population-based RI are not appropriate for monitoring since a significant change in an individual may go unnoticed. In such cases the reference change value (RCV), which is the significant change between two samples taken from the same individual at different points of time, is more suitable [7]. Studies on biological variation are needed to determine whether population-based RI are appropriate to use for HOMA-IR and fasting insulin.

The objectives of this study were to (i) evaluate associations between body fat, HOMA-IR and fasting insulin, (ii) determine population-based RI of HOMA-IR in lean cats, and (iii) evaluate the biological variation of HOMA-IR and fasting insulin in cats.

## Results

### Descriptive statistics

**Fig. 1:**
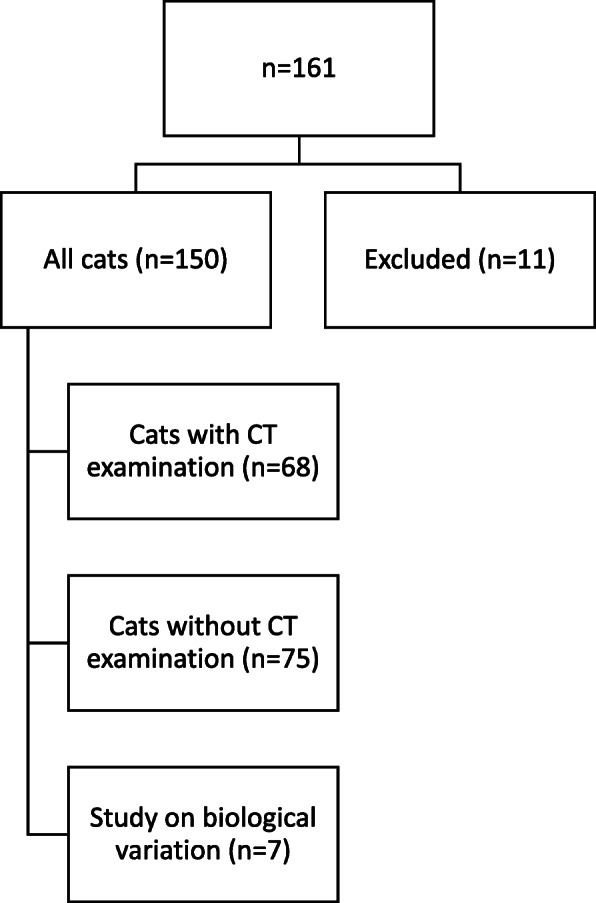
Flowchart of study design for measurement of fasting serum insulin and glucose. Of the 161 cats enrolled in the study, 11 were excluded. The remaining 150 cats included one group of 68 cats that had body fat percentage evaluated by computed tomography (CT), one group of 75 cats without CT examination, and one group of 7 cats sampled multiple times for evaluation of biological variation

Nine of 161 cats were considered stressed at sampling and were excluded from further analyses. Four of the remaining 152 cats had blood glucose > 10 mmol/L. One of these cats had elevated fructosamine and was excluded due to a subsequent diagnosis of DM. The other three cats had fructosamine concentrations within RI and at follow-up two years later none had developed DM according to the owners. One cat had extremely high insulin concentration, which was not linear upon dilution. This cat was suspected to have interfering antibodies and was excluded [8]. Flowchart of study design is presented in Fig. 1.

Mean age in the remaining 150 cats was 7.1 (SD 3.8) years. Of cats classified using the body condition score (BCS) 5 grade scale (*n* = 20) 15 were graded 3/5 and five were graded 4/5. Of cats classified according to the 9 grade scale (*n* = 130) one was graded 3/9, six graded 4/9, 51 graded 5/9, 33 graded 6/9, 26 graded 7/9, 10 graded 8/9 and three graded 9/9. There were 72 males (67 castrated and 5 intact) and 78 females (55 castrated and 23 intact). Breeds included Domestic short- and longhair (*n* = 53), Maine Coon (*n* = 37), Birman (*n* = 23), Burmese cats (*n* = 14), British shorthair (*n* = 5), Norwegian forest cat (*n* = 4), Siberian cat (*n* = 4), Ragdoll (*n* = 2), Sphynx (*n* = 2), Cornish Rex (*n* = 2), Balinese (*n* = 1), Bengal (*n* = 1), European shorthair (*n* = 1) and Ocicat (*n* = 1). A subgroup of 68/150 cats was used for determination of body fat percentage (BF%) by computed tomography (CT). Mean age of this subgroup was 8.2 (SD 3.3) years and these cats consisted of Domestic short- and longhair (*n* = 40), Maine Coon (*n* = 11), British shorthair (*n* = 5), Norwegian forest cat (*n* = 4), Siberian cat (*n* = 2), Ragdoll (*n* = 2), Sphynx (*n* = 2), European shorthair (*n* = 1) and Ocicat (*n* = 1). There were 39 males (all castrated) and 29 females (25 castrated and 4 intact). BF% ranged from 15.3 to 61.9% (mean 38.0, SD 11.9).

### Evaluation of BCS in relation to BF% and HOMA-IR, insulin and glucose concentrations in lean and overweight cats

One of the 15 BCS lean cats was classified as overweight based on BF% (35 ≤ BF% < 45) which yielded a negative predictive value (NPV) of 93.3% for identifying lean cats by BCS using BF% as reference method. The remaining 53 cats were BCS ≥ 6 and of these 14 were lean based on BF% (< 35%), yielding a positive predictive value (PPV) of 73.6% for identifying overweight/obese cats by BCS using BF% as reference method. Most of the later erroneously graded cats were clinically considered only mildly overweight (12 cats BCS 6/9, and 2 cats BCS 7/9). Distribution of BCS in relation to BF% is shown in Table 1. HOMA-IR, insulin and glucose concentrations were significantly higher in overweight cats than lean cats (p < 0.0001, p = 0.0002 and p < 0.0001), respectively). Descriptive statistics stratified by BCS are presented in Table 2.

**Table 1:** Descriptive statistics of serum concentrations of glucose and insulin, and HOMA-IR in 68 cats stratified in to lean, overweight and obese groups according to percentage body fat (BF%)

	Lean BF% < 35 (*n* = 28) ^a^		Over-weight 35 ≤ BF% < 45 (*n* = 21)^b^		Obese BF% ≥ 45 (*n* = 19)^c^	
	Mean (SD)	Median (Q1, Q3)	Mean (SD)	Median (Q1, Q3)	Mean (SD)	Median (Q1, Q3)
HOMA-IR	1.3 (0.9)	1.0 (0.6,2.1)	2.3 (1.9)	1.8 (0.9,2.9)	2.9 (1.6)	2.7 (1.8,4.1)
Insulin (mU/L)	4.9 (3.5)	3.6 (2.5,7.4)	7.0 (3.2)	7.2 (3.7,9.7)	9.8 (4.8)	9.3 (6.8,12.5)
Glucose (mmol/L)	6.1 (1.2)	5.8 (5.4, 6.9)	6.8 (2.9)	5.8 (5.2, 7.5)	6.6 (1.2)	6.3 (5.6, 7.5)

^a^BCS 5 (*n* = 14); BCS 6 (*n* = 12), BCS 7 (*n* = 2)

^b^BCS 5 (*n* = 1); BCS 6 (*n* = 6), BCS 7 (*n* = 12), BCS 8 (*n* = 2)

^c^BCS 6 (*n* = 1), BCS 7 (*n* = 8), BCS 8 (*n* = 8), BCS 9 (*n* = 2)

**Table 2:** Descriptive statistics of serum glucose, insulin and HOMA-IR in 150 cats classified as lean or overweight according to body condition score (BCS)

	Lean (BCS ≤ 5) *n* = 73		Overweight (BCS ≥ 6 ) *n* = 77		Wilcoxon Rank Sums test
	Mean (SD)	Median (Q1, Q3)	Mean (SD)	Median (Q1, Q3)	*p*-value
HOMA-IR	1.1 (0.9)	0.8 (0.4, 1.4)	2.0 (1.6)	1.4 (0.9, 2.7)	< 0.0001
Insulin (mU/L)	4.7 (3.8)	3.3 (2.3, 5.5)	6.7 (4.1)	6.3 (3.6, 8.8)	0.0002
Glucose (mmol/L)	5.2 (1.7)	4.8 (4.3, 5.6)	6.3 (2.0)	5.8 (5.1, 7.1)	< 0.0001

### Association between BF%, HOMA-IR, insulin and glucose concentrations

Descriptive statistics for HOMA-IR, insulin and glucose concentrations in relation to BF% are presented in Table 1. There were significant associations between BF% and ln(HOMA-IR) and between BF% and ln(Insulin) (*p* = 0.0010 and *p* = 0.0017, respectively, Table 3). A 10% increase in BF% was associated with an average increase of HOMA-IR and insulin by 30% and 25%, respectively. The BF% had no significant association with blood glucose (*p* = 0.12). Whether the cat had signs attributed to the locomotor apparatus or not was considered a potential additional explanatory factor in the regression of ln(HOMA-IR) or ln(Insulin) on BF(%), but this factor was not significant (*p* = 0.2 and *p* = 0.4, respectively) and cats with signs from the locomotor apparatus were included.

**Table 3:** Univariate linear regression with body fat percentage as predictor in 68 cats

Response variable	Beta (coefficient)	Rsq (%)	*p*-value
ln(HOMA-IR)	0.0262	0.15	0.0010
ln(Insulin)	0.0225	0.13	0.0017
ln(Glucose)	0.004	0.04	0.12

### Biological variation of HOMA-IR and fasting insulin

**Fig. 2:**
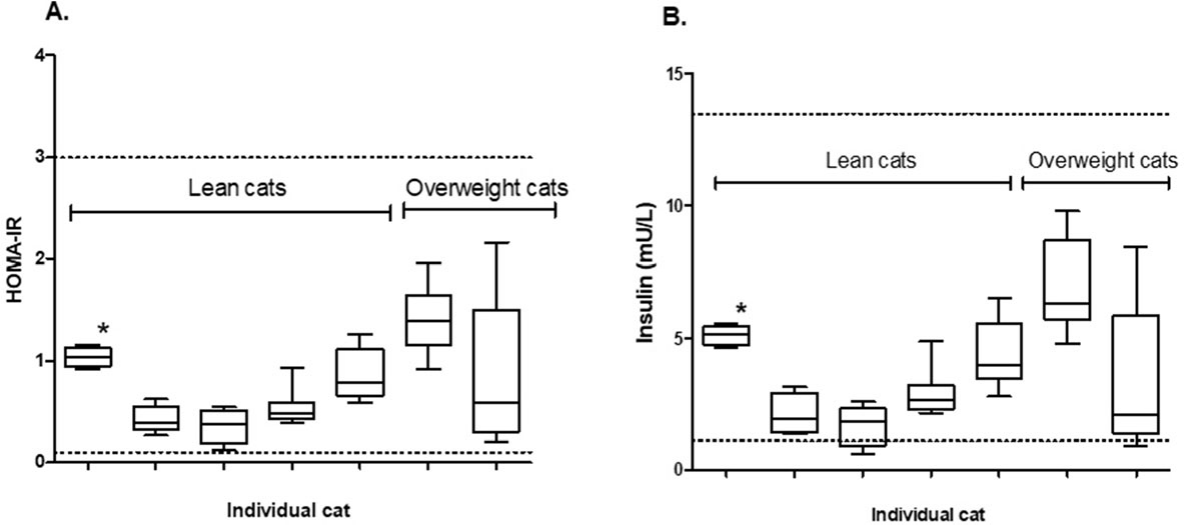
Biological variation of HOMA-IR (**a**) and serum insulin concentrations (**b**) in lean and overweight cats (*n* = 7) sampled once a week for 5 weeks. Whiskers indicate min and max values. Dotted line represents population-based reference interval. Asterix (*) denotes one duplicate outlier excluded

One cat had unmeasurable high insulin concentrations at one sample occasion and was classified as an outlier, and in one cat there was a pipetting error during analysis leaving only one replicate available for that sampling occasion. Indices of biological variation are presented in Table 4. Five of the seven cats sampled for biological variation were classified as lean (BCS 3/5) and two cats classified as overweight (BCS 4/5). The two overweight cats reached the highest HOMA-IR and insulin concentrations though not statistically classified as outliers (Fig. 2). When the two overweight cats were excluded coefficient of variation between cats (CV_G_) was similar but coefficient of variation within a cat (CV_I_) was considerably lower (Table 4).

**Table 4:** Indices of biological variation of fasting insulin concentrations and HOMA-IR in 7 cats (5 lean (BCS = 3/5) and 2 overweight (BCS = 4/5)) sampled once a week for 5 weeks

	HOMA-IR All cats (*n* = 7)	HOMA-IR Lean cats (*n* = 5)	Insulin (mU/L) All cats (*n* = 7)	Insulin (mU/L) Lean cats (*n* = 5)
Mean	0.78	0.63	3.8	3.2
Range	0.1–2.2	0.1–1.3	0.6–9.8	0.6–6.5
CV_G_ % (95% CI)	52.3 (0-87.7)	51.0 (0-90.1)	54.4 (0-91.2)	52.1 (0-92.1)
CV_I_ % (95% CI)	51.0 (33.4–65.4)	38.2 (22.1–50.2)	48.7 (32.1–62.3)	38.0 (22.1–49.9)
CV_A_ % (95% CI)	7.4 (5.9–9.7)	7.4 (5.8–10.3)	6.6 (5.3–8.6)	6.6 (5.2–9.2)
RCV increase (%)	385	284	364	281
RCV decrease (%)	26	35	27	36
II	1.0	1.31	1.11	1.35

*CV*_G_indicates between-cat coefficient of variation; *CV*_I_ within-cat coefficient of variation; *CV*_*A*_ analytical coefficient of variation derived from the mixed model analysis; *RCV* reference change value based on $RCV \left(\%\right)=100 \text{e}\text{x}\text{p}(\pm 1.96 \sqrt{ 2\left({SD}_{I}^{2}(\text{ln}x\right)+{SD}_{A}^{2}(\text{ln}x\left)\right)}$ ; II, index of individuality based on CV_G_/(CV_I_^2^ + CV_A_^2^)^0.5^

### Reference interval for HOMA-IR and insulin concentrations

For RI calculations, cats with signs from the locomotor apparatus were excluded, and based on the high NPV, cats that did not have BF% determined by CT were classified as lean when they had BCS ≤ 5 (scale 1–9) or BCS ≤ 3 (scale 1–5). For cats sampled multiple times to determine biological variation, the sampling occasion used for RI calculation was selected randomly. This resulted in a group of 61 cats for RI calculations. For HOMA-IR and insulin, one of the 61 cats was considered an outlier according to Tukey’s test and this cat was excluded from the RI calculation. The population consisted of Maine Coon (*n* = 17), Birman (*n* = 20), Domestic short- and longhaired cats (*n* = 10), Burmese (*n* = 8), Siberian cat (*n* = 2), Cornish Rex (*n* = 1), Bengal (*n* = 1) and Balinese (*n* = 1). The RI was obtained after Box-Cox transformation and using the robust method [9], which down-weighs data far from the central location. The RI in the 60 cats was 0.1–3.0 for HOMA-IR (90% CI for lower limit 0.1–0.2, upper limit 2.4–3.6) and for insulin 0.7–12.1 mU/L (90% CI for lower limit 0.5–1.0, upper limit 9.8–14.8). The 90% CI of the upper limits of the RI in both HOMA-IR and insulin RI were wider than recommended [10].

## Discussion

Obesity in cats may cause IR with higher circulating insulin concentrations required to achieve normoglycemia [2]. In the present study we calculated HOMA-IR and measured fasting insulin and found that both were positively associated with BF%, suggesting IR with increasing fat percent. A similar association between HOMA-IR and obesity has previously been reported [11]. We speculated that the HOMA-IR model, which takes into consideration both insulin and glucose concentrations, would have a stronger association with BF% than insulin alone. This was not the case, instead we found a similar association for both HOMA-IR and insulin to BF% which is in line with results reported by Appleton et al. [5]. In the study by Appleton et al. HOMA-IR was evaluated in relation to the minimal model analysis and in overweight cats with IR there was a stronger correlation to the minimal model analysis compared to a group with mixed weight cats. It is possible that HOMA-IR is most useful to detect moderate to severe IR and not mild changes. Ideally, HOMA-IR should be evaluated to the euglycemic clamp, which is considered the gold standard method for evaluating IR. This was not performed in our study and to our best knowledge, no data investigating this association has been published. Even though HOMA-IR has been used in previous feline studies [5, 11, 12] it is possible that the mathematical formula is not optimal for cats in determining IR, which may explain why HOMA-IR did not have a much stronger association with BF% than fasting insulin. Another factor that may affect the usefulness of HOMA-IR in cats is stress-related hyperglycemia. In one study, cats demonstrated significant increase in blood glucose after a spray bath [13], indicating that stress potentially could increase HOMA-IR. The same study also showed a trend towards increased insulin concentration after stress, which successively decreased although statistical significance was not demonstrated for the increase in insulin concentration [13]. To avoid influence of stress on HOMA-IR, cats that did not have sufficient cooperative abilities were excluded in the present study. Nevertheless, some stressed cats may have appeared calm despite being stressed and thus would not have been identified, so it cannot completely be excluded that stress could have influenced results in the present study. In the sampled population, two lean animals had blood glucose of 13 mmol/L with fructosamine concentrations within RI. The cause of hyperglycemia in these cats is unknown however IR or undetected stress-related hyperglycemia is possible. There is scarce information about the effects of stress-related hyperglycemia and its effect on insulin secretion and HOMA-IR. This needs further attention in future studies.

Cats with signs from the locomotor apparatus at clinical examination were excluded for calculations of RI but kept in the other statistical analyses as we could not demonstrate a significant effect on HOMA-IR or insulin when added into the regression analyses. Links between IR and osteoarthritis are seen in humans [14] and in diabetic people there is evidence of accelerated cartilage degeneration [15]. A similar scenario is possible in the cat and investigations of derangement in glucose metabolism as a risk factor for development of osteoarthritis in cats requires further studies. Due to the common occurrence of osteoarthritis in cats [16], the sampled population is likely to be representative of cats seen in general practice.

In humans HOMA-IR has been used to estimate IR in DM type 2 [17]. Cats with DM often present with DM similar to type 2 in humans, but may have low insulin concentrations at diagnosis [18–20], likely due to glucotoxicity [21]. Low insulin as a result of impaired beta-cell function together with high glucose may give incorrect interpretation of HOMA-IR in diabetic cats, hence HOMA-IR as an indicator of IR is likely most useful when adequate insulin secretion is still present. Since most cats develop DM type 2 the use of HOMA-IR in obese cats and its relation to development of DM is interesting and future studies are needed to evaluate the clinical value of HOMA-IR as predictor for DM.

It may be difficult to compare RI for an analyte if different assays are used and no certified reference material or gold standard method is available. Results may for example differ due to different cross-reactivity of antibodies or material used for calibration. There is no certified reference material or gold standard method when measuring feline insulin, thus insulin concentrations may vary depending on the assay used. Nevertheless, a previous study reported RI for HOMA-IR based on 25 cats to be 0-2.84 [5], which is similar to our results. Appleton et al. [5] discussed that cats with HOMA-IR above the mean of their RI may be at risk for developing IR and DM, and that early detection of such individuals could allow for preventive actions to be taken. The cause of IR is multifactorial [22] and in the study by Appleton et al. [5] some lean cats were IR and some obese cats were not. Since this was a retrospective study, we did not evaluate IR by intravenous glucose tolerance tests. It cannot be excluded that some lean cats could have been IR and if so this may have contributed to a falsely high RI.

With the formulas used to calculate index of individuality (II), a value < 0.7 indicates that population-based RI are useful, whereas a value > 1.7 should raise a concern that a significant change in an individual may not be detected if using population-based RI. Index of individuality for insulin and HOMA-IR in lean cats was 1.0-1.35, which indicates intermediate individuality and population-based RI may be of limited use. Thus, it may be valuable to use RCV for monitoring cats at risk for developing IR even if HOMA-IR or insulin concentrations are within the population-based RI. A human study reported increasing CV_I_ of fasting insulin with deteriorating glucose tolerance [23] and interestingly two overweight cats in the study had similar results and showed the widest range of HOMA-IR and insulin. Biological variation studies of higher numbers of cats can help to investigate whether overweight cats, like humans with poor glucose tolerance, truly have higher variations in HOMA-IR and insulin concentrations. In addition, adding more cats would decrease the width of the CI of the RIs, which were wide in the present study. However, conducting studies of biological variation is challenging since all samples need to be analysed together to exclude between-assay variation. In the present study, where samples were analysed in 96-well plates using enzyme-linked immunosorbent assay (ELISA), the number of included animals and sample occasions were limited to the number of wells.

In the formulas relating to biological variation we used analytical coefficient of variation (CV_A_) derived from the random-effects model analysis. Since all samples were analysed at once, this CV_A_ included only within-run CV and may therefore be lower than expected for clinical samples. At the laboratory used in this study inter-assay CV for serum insulin and glucose were reported to vary between 7.6–14% and 0.7–1.1%, respectively. CV_A_ derived from the random-effects model analysis was slightly lower, which may have caused a minor error. For example, the laboratory reported the highest CV_A_ of 14% at low insulin concentrations. A healthy cat with insulin concentrations of 2 mU/L will need concentrations above 7.3 mU//L at the second sampling to be considered abnormal if using CV_A_ of 6.6%. With CV_A_ of 14% an increase above 7.6 mU/L would be considered abnormal. Note that the population-based RI was 0.7–12.1 mU/L and would not have identified this cat as abnormal. Ideally, total CV_A_ established by each laboratory should be used when calculating for example RCV, and for HOMA-IR, which is based on both insulin and glucose measurements, one should be aware that two analytical CVs need to be considered. When the analyte needs to be transformed to the logarithmic scale a formula to reverse the transformation must be used to calculate RCV. This back-transformation makes the calculation slightly more complicated, but has the strong advantage of allowing the significant increase to be larger than the significant decrease.

In the present study the results from BCS were compared to BF% estimated by CT. A previous feline study evaluated the nine scale BCS system [24] for assessment of body composition [25]. In that study, dual energy x-ray absorptiometry was used to determine BF%, and although there was some overlap in BF% between the BCS categories, the results suggested that BCS is useful for assessing body fat in cats [25]. Computed tomography has been shown to give results on body composition in cats in close agreement to those obtained with dual energy x-ray absorptiometry scans [26]. A limitation of the current study is that there were three veterinarians evaluating BCS and two different scales were used. Due to the use of two scales cats were only grouped as overweight or not in statistical analyses. Some cats in the present study were classified as overweight based on BCS, but were not overweight according to BF%. This may have affected results where the BCS was used for grouping, since some lean cats (based on BF%) may have been included in the overweight group (based on BCS), however it less likely that overweight cats were included in the lean population used for RI calculations.

## Conclusions

In summary we found that HOMA-IR and fasting insulin were associated with body fat. We established population-based RI for HOMA-IR as well as indices of biological variation for HOMA-IR and fasting insulin, which may be used when interpreting HOMA-IR and fasting insulin in cats. Further studies to determine if high HOMA-IR and fasting insulin are associated with the development of DM are needed.

## Methods

### Animals

In this retrospective study, sera from 161 privately-owned cats involved in method validation and ongoing feline obesity and osteoarthritis studies were included (Fig. 1) [6, 27–29]. To recruit cats to the studies flyers were given at cat exhibitions, advertisement done at entrances to animal hospitals and shopping centers as well as on social media, and e-mails sent to university students and staff at animal hospitals asking them to inform clients about the projects. Except for a subgroup of cats, which were permitted to have gait abnormalities, inclusion criteria were that owners must consider their cat to be healthy and that cats were considered healthy at the veterinary clinical examination. Cats were excluded if they were < 1 year old, pregnant or non-fasted. The studies were approved by Uppsala Ethics Committee on Animal Experiments (no. C22/9, C282/11, C299/12, C27/14, C12/15, C23/15, C102714/15) and by the Swedish Board of Agriculture (31-1364/09 and 31-11654/12). Cats sampled at the animal hospitals returned home with the owner the same day. Clinical examination was performed by three veterinarians, and apart from a subgroup of cats (*n* = 33) participating in the osteoarthritis study, which had signs attributed to the locomotor apparatus, all cats were considered clinically healthy. Clinical examination included cardiac and lung auscultation, abdominal palpation, palpation of lymph nodes, abdominal palpation, visual inspection of genital area, oral examination and general condition. In a subgroup of cats (*n* = 33) there was also an orthopedic exam (evaluation of gait abnormalities, range of motion, joint effusion, joint pain, crepitus, periarticular thickening). Evaluation of BCS was performed using a 1– 9 or 1–5 scale [25, 30] and BF% were determined by whole body CT (*n* = 68) [26]. Based on the findings on clinical examination cats were subdivided into the groups lean (BCS ≤ 5 using scale 1–9 and ≤ 3 using scale 1–5) or overweight (BCS ≥ 6 using scale 1–9 and ≥ 4 using scale 1–5).

### Blood sampling and analytical procedures

Cats had blood samples taken either in their home environment, at the University Animal Hospital, Uppsala or at Bagarmossen Animal Hospital, Stockholm, Sweden. Cats were allowed a calming down period after arrival at the hospital before the clinical examination. Blood samples were collected after the clinical examination.

Seven of the cats (all healthy adults) were sampled once weekly for five weeks for a biological variation study where biological variation of glucose was reported [29]. The cats in the biological variation study were sampled in their home environment during the morning after an overnight fast (~ 12 h) and the clinical examination and blood sampling from the cephalic vein were done by the same veterinarian. All samples were centrifuged after 30–60 minutes at 3000x*g* for 5 minutes using the same centrifuge (EBA20, Andreas Hettich GmbH & Co. KG. Tuttlingen, Germany) each time. Sera was either frozen to -80ºC the same day or first frozen to -20ºC and then transferred to -80ºC within a week.

All other cats (*n* = 143) were fasted for at least 8–12 h prior to sampling. Blood was collected from the cephalic or jugular vein and placed into serum tubes, centrifuged after 30–60 minutes and sera either frozen to -80ºC the same day or first frozen to -20ºC and then transferred to -80ºC within a week. Sera was kept at -80ºC until analysis and thawed up to three times before analysis. Serum insulin concentrations have been shown to be stable for at least four and glucose concentrations for up to ten freeze-thawing cycles [6, 31]. Glucose was analysed in random order using hexokinase/glucose-6-phosphate dehydrogenase (Glucose, Architect cSystems, Abbott Diagnostics, Illinois) with a biochemistry analyzer (Architect c4000, Abbott Diagnostics, Illinois). Intra- and interassay coefficients of variation (CV) for glucose were both reported by the laboratory to be < 1.1%. In cats with blood glucose > 10 mmol/L fructosamine concentrations were analysed as an aid in distinguishing stress-related hyperglycemia from DM. Fructosamine was measured by the nitrotetrazolium blue-method (ABX Pentra, Horiba group, Montpellier, France) using a standard biochemistry instrument (Architecht c4000, Abbott Diagnostics, Illinois). Intra- and interassay CV for fructosamine were both reported by the laboratory to be < 1.6%. Cats that were not cooperative and could not be manually restrained for the blood sampling were excluded. Cats with glucose concentration > 10 mmol/L and fructosamine concentrations within RI were followed up after two years by telephone contact with the owners to record health status.

Insulin was measured by a previously validated feline ELISA with intra- and interassay CV reported to be 2.0–4.2% and 7.6–14%, respectively [6]. Concentrations of insulin were given in ng/L and were multiplied by 0.023 for conversion to mU/L according to the manufacturer’s instructions. Samples analysed to determine biological variation had been thawed twice and were analysed in duplicates in random order on one plate.

Insulin resistance was estimated by calculation of HOMA-IR as glucose (mmol/L) x insulin (mU/L)/22.5, as described by Matthews et al. [17, 32] and previously used in cats [5, 11, 12].

### Determination of BF%

Sedation and whole body CT was performed after clinical examination and blood sampling. The cats were sedated with medetomidine hydrochloride (Sedator®, 1 mg/ml, Dechra Veterinary Products, Lostock Gralam, United Kingdom) or with medetomidine hydrochloride and butorphanol tartrate (Dolorex®, 10 mg/ml, Intervet Inc., Stockholm, Sweden) and positioned in sternal recumbency in an extended position. A 64-slice CT scanner (Definition, Siemens Medical Systems, Erlangen, Germany) using a helical protocol was used with a slice thickness and increment, 0.6 mm; tube voltage, 250 kVp; tube current, 160 mA; soft tissue convolution kernel, B30f; focal spot, 1.2 mm; reconstruction diameter was adjusted individually according to the cats size. The CT images were transferred to a Digital Imaging and Communications in Medicine workstation (Horos, version 2.4.0., https://www.horosproject.org) where manual segmentation of the images was done by a Diplomate of the European College of Veterinary Diagnostic Imaging (CJL) using the ‘freehand pencil tool’ and the ‘generate missing regions of interest (ROI) tool’. For the segmentation window width 400 Hounsfield Units (HU) and window level 40 HU were used, and the urine in the urinary bladder, the table and any objects external to the cats body with HU values between − 251 HU and 251 HU were removed from the images (HU values set to -1024 HU). The BF% was calculated according to a published method [26]. Briefly, ImageJ software (1.44o, 64-bit, National Institutes of Health, USA) was used to generate a frequency data list of the HU voxel values in the whole body CT image. All voxels ​​in the range of -250 to + 250 HU were selected and copied to Microsoft excel (2013, Microsoft, USA), where a frequency histogram with fat attenuation and lean soft tissue attenuation peaks was generated and the mid-point between these two peaks was calculated. Voxels with HU values ≥ -250 HU and ≤ the mid-point HU value between the two peaks in the histogram were considered to be fat attenuation, and voxels with HU values > than the mid-point HU value between the two peaks in the histogram and ≤ 250 HU were considered to be lean soft tissue attenuation. The BF% was calculated by the equation; number of fat voxels divided by the sum of the numbers of fat and lean soft tissue voxels and that figure was multiplied by 100. Cats were classified as lean (BF% < 35%), overweight (35 ≤ BF% < 45) or obese (BF% ≥ 45) according to Bjornvad et al. [11].

### Statistical analyses

JMP was used for statistical analyses (Version Pro 14, SAS Institute Inc., Cary, NC). Differences of HOMA-IR and insulin between lean and overweight cats were investigated by the Wilcoxon rank-sum test. The power of this test is a function of the proportion, ${p}^{{\prime }{\prime }}$, of pairs of observations for which $X<Y$, where $X$ and $Y$ are random observations from the first and the second group, respectively [33]. With sample sizes of 73 and 77, the power is 80% to detect ${p}^{{\prime }{\prime }}=0.63$ at significance level 5%. For evaluation of associations between BF% and HOMA-IR, insulin and glucose concentrations linear univariate regression was used. Residuals were evaluated for normal distribution by histograms and Q-Q-plots. Preliminary prediction models demonstrated non-normality of the residuals and HOMA-IR, insulin and glucose were therefore transformed to the natural logarithmic scale. *P*-values < 0.05 were considered significant.

For the study of biological variation unbounded restricted maximum likelihood (REML) was used for estimation of variance components. A random-effects model with random effects of cats and samples, where samples were nested within cats, was fitted. Since preliminary analyses demonstrated skewed distributions HOMA-IR and insulin were log-transformed before analysis, using the natural logarithm. Standard deviation on the logarithmic scale, $\text{S}\text{D}(\text{ln}x)$, derived from the random-effects model analysis was used to calculate CV using the equation provided by Cole [34]:

$$\text{C}\text{V}= \sqrt{\text{exp}\left({\text{S}\text{D}}^{2}\left(\text{ln}x\right)\right)-1}$$

This random-effects model analysis yielded three variance components, which expressed as CVs were denoted CV_G_, CV_I_, and CV_A_, for variation between cats, within cats, and between duplicates, respectively. Wald 95% confidence intervals (CI) for the variance components were computed and expressed as CVs, using the Cole [21] equation, where negative lower limits were set to zero. To evaluate the use of population-based RI, index of individuality (II) was calculated as [35]:

$$\text{I}\text{I}=\frac{\text{C}{\text{V}}_{\text{G}}}{\sqrt{\text{C}{\text{V}}_{\text{I}}^{2}+\text{C}{\text{V}}_{\text{A}}^{2}}}$$

Using this formula, II < 0.7 indicates low individuality and appropriate use of population-based RI, whereas II > 1.7 indicates high individuality and the need for RCV when interpreting results [22]. RCV indicates the statistical significant change between serial measurements of an individual. Since a bidirectional change in concentrations was considered important a two-sided formula with 95% probability for ln-normal distributed data was used. With this approach RCV will not be symmetrical, i.e. the significant increase will be larger than the significant decrease. RCV was computed as:

$$\text{R}\text{C}\text{V} \left(\%\right)=100 \text{e}\text{x}\text{p}\left\{\pm 1.96 \sqrt{ 2\left({\text{S}\text{D}}_{\text{I}}^{2}(\text{ln}x\right)+{\text{S}\text{D}}_{\text{A}}^{2}(\text{ln}x\left)\right)}\right\}$$

where ${\text{S}\text{D}}_{\text{I}}^{2}\left(\text{ln}x\right)$ and ${\text{S}\text{D}}_{\text{A}}^{2}\left(\text{ln}x\right)$ are estimates of within cats and between-duplicate variance, respectively, on the log scale [36].

Reference intervals for HOMA-IR and insulin were determined in lean healthy cats, excluding cats with signs attributed to the locomotor apparatus, by using the software program Reference value advisor [9], where Tukey´s rule (more than 1.5 times the interquartile range from the quartiles) was used for identifying outliers. The 90% CI of the upper and lower limits were calculated using bootstrapping, which was default in the software program [9].

## Data Availability

The datasets used during the current study are available from the corresponding author on request.

## Abbreviations

BCS: Body condition score
BF%: Body fat percentage
CI: Confidence interval
CT: Computed tomography
CVA: Analytical coefficient of variation
CVG: Between-cat coefficient of variation
CVI: Within-cat coefficient of variation
DM: Diabetes mellitus
HOMA-IR: Homeostasis model assessmen.
HU: Hounsfield units
II: Index of individuality
NPV: Negative predictive value
PPV: Positive predictive value
IR: Insulin resistance
RCV: Reference change value
REML: Restricted maximum likelihood
RI: Reference interval

## Authors’ information (Relevant information about the authors (competence))

EMS – MScVetMed, PhD, Dipl. ECVCP (European College of Veterinary Clinical Pathology); CJL - BVSc, MVetMed, PhD, Dipl. ECVDI (European College of Veterinary Diagnostic Imaging); JF – PhD, senior statistician; MÖ – MScVetMed, PhD; SS – MScVetMed, PhD; AB – MScVetMed, PhD, Dipl. ECVSMR (European College of Veterinary Sports Medicine and Rehabilitation); CL – MScVetMed, PhD, Dipl. ECVP (European College of Veterinary Pathologists).
